# Morphological and molecular characterization of *Filenchus pseudodiscus* n. sp. from east Golestan province, north Iran; with an updated phylogeny of *Malenchus* Andrássy, 1968 (Tylenchomorpha: Tylenchidae)

**DOI:** 10.21307/jofnem-2021-069

**Published:** 2021-08-05

**Authors:** Parnaz Mortazavi, Fariba Heydari, Joaquín Abolafia, Pablo Castillo, Majid Pedram

**Affiliations:** 1 Department of Plant Pathology, Faculty of Agriculture, Tarbiat Modares University, Tehran, Iran; 2 Departamento de Biología Animal, Biología Vegetal y Ecología, Universidad de Jaén, Campus Las Lagunillas, s/n, 23071, Jaén, Spain.; 3 Instituto de Agricultura Sostenible (IAS), Consejo Superior de Investigaciones Cientíﬁcas (CSIC), Avenida Menéndez Pidal s/n, 14004, Córdoba, Spain.

**Keywords:** LSU rDNA D2-D3, Morphology, New species, Phylogeny, SSU rDNA, Taxonomy

## Abstract

During a survey in Golestan province, north Iran, two populations belonging to the family Tylenchidae were recovered in association with *Quercus* sp., and a rotten wood sample of an unidentified forest tree. The first recovered species was mainly characterized by having a disc-like differentiation in the frontal end under the light microscope (LM), proposing it as a tentative member of the genus *Discotylenchus*. Detailed morphological studies using scanning electron microscope (SEM), however, did not reveal a true disc, but showing the smooth cephalic region, and a narrow annulus behind the cephalic plate. Based upon the cephalic region structure, and by lacking a true disc, the species was identified as a member of the genus *Filenchus*. This population was further characterized by 555 to 618 μm long females, lateral fields with four incisures, 9 to 10 μm long stylet, spermatheca large, including spheroid sperm, post-vulval uterine sac (PUS) 8 to 12 µm long and gradually tapering to an elongate conoid tail with pointed tip. It was compared with relevant species of *Filenchus* having four incisures in the lateral fields and similar general morphology. By having a disc-like differentiation in the frontal end under the LM, it was further compared with three similar known species of *Discotylenchus*. The morphological comparisons with species under two aforementioned genera showed the recovered population belongs to an unknown species, described herein as *Filenchus pseudodiscus* n. sp. The molecular phylogenetic relationships of the new species using partial small and large subunit ribosomal RNA gene (SSU and LSU D2-D3 rDNA) sequences were reconstructed and discussed. *Malenchus gilanensis*, the second recovered and studied species was originally established based upon traditional criteria. An updated LSU phylogeny of the genus *Malenchus* by including *M. gilanensis* was also presented and its results were discussed.

According to Geraert (2008), the subfamily Tylenchinae (Örley, 1880), in the family Tylenchidae (Örley, 1880), currently includes 15 genera. The genus *Labrys* (Qing and Bert, 2018) represents the last genus added to the subfamily. The genus *Filenchus* (Andrássy, 1954) *sensu lato*, represents the largest genus and currently includes 94 species. *Discotylenchus* (Siddiqi, 1980) currently includes six species (Geraert, 2008) and *Malenchus* (Andrássy, 1968) harbours 36 valid species under two subgenera *Malenchus* (*Malenchus*) (Andrássy, 1968) and *Malenchus* (*Telomalenchus*) (Geraert, 2008; Qing and Bert, 2017; Siddiqi, 2000). There are currently available molecular data for representatives of *Filenchus* and *Malenchus* (e.g. Atighi et al., 2013; Qing et al., 2017). The genus *Discotylenchus* was originally established based upon traditional criteria, and SEM and molecular data of the type populations of its six known species *sensu* Geraert (2008) are not available. The only available SEM images of the genus correspond to an Iranian population of *Discotylenchus discretus* (Siddiqi, 1980) by Yaghoubi et al. (2016). The molecular phylogenetic studies on *Filenchus* spp. and *Malenchus* spp. were however the subject of several recent studies (Atighi et al., 2013; Bai et al., 2020; Pedram et al., 2018; Qing and Bert, 2017; Qing et al., 2017).

During recent years, several taxonomic studies were performed on Tylenchidae in Iran (e.g. Gharahkhani et al., 2020; Hosseinvand et al., 2020; Panahandeh et al., 2019a, b). In the present study, two populations of the family Tylenchidae were recovered from natural forests of Golestan province, north Iran. The first species appeared as being a new member of *Filenchus*, and the second species belonged to *Malenchus gilanensis* (Jalalinasab et al., 2019). Thus, the present study aims to (i) characterize the new species using both traditional and molecular criteria, and (ii) update the phylogeny of *Malenchus* by including *M. gilanensis* in the LSU tree.

## Materials and methods

### Sampling, nematode extraction, mounting, and drawing

A total of 50 soil, and 36 rotten wood samples were collected from the natural forests in Golestan province, north Iran, during 2017 and 2018. The samples were placed in plastic bags, transferred to the nematology laboratory of Tarbiat Modares University and maintained at cool temperature condition. Nematodes were extracted from samples using the tray method (Whitehead and Hemming, 1965), heat killed by adding boiling 4% formalin solution and transferred to anhydrous glycerin according to De Grisse (1969). Drawings and morphological studies were performed using a drawing tube attached to a Nikon E600 light microscope; and were redrawn using CorelDraw software version 2012. The light microphotographs of the fresh individuals and mounted specimens were prepared using an Olympus BX51 microscope, equipped with a digital DP72 camera (Olympus) and differential interference contrast (DIC) optics.

### Scanning electron microscopy

Four mounted female specimens of the new species were selected for observation under SEM following the protocol of Abolafia (2015). The nematodes were hydrated in distilled water, dehydrated in a graded ethanol and acetone series, critical point dried, coated with gold, and observed with a Zeiss Merlin scanning electron microscope (Carl Zeiss, Germany).

### DNA extraction, polymerase chain reaction (PCR), and sequencing

DNA was extracted from four female specimens of the both recovered populations by squashing each specimen in 15 µl TE buffer (10 mM Tris-Cl, 0.5 mM EDTA; pH 9.0, Qiagen) (four DNA samples were prepared for each species) after their examination on temporary slides. DNA samples were stored at ‒20°C until used as PCR templates. Partial sequence of the SSU rDNA gene was amplified using primers 988F (5′-CTCAAAGATTAAGCCATGC-3′), 1912R (5′-TTTACGGTCAGAACTAGGG-3′), 1813F (5′-CTGCGTGAGAGGTGAAAT-3′) and 2646R (5′-GCTACCTTGTTACGACTTTT-3′) with resulting PCR products ranging from 890 to 930 and 970 to 1,017 bp, respectively (Holterman et al., 2006). The forward primer D2A (5′-ACAAGTACCGTGAGGGAAAGTTG-3′) and reverse primer D3B (5′-TCGGAAGGAACCAGCTACTA-3′) (Nunn, 1992) were used for amplification of D2-D3 expansion segments of LSU rDNA. The thermocycling program for amplification of both loci was as follows: denaturation at 95°C for 4 min, followed by 32 cycles of denaturation at 94°C for 30 sec, annealing at 52°C for 40 sec, and extension at 72°C for 80 sec. A final extension was performed at 72°C for 10 min. The PCR products were sequenced using the same primers used for their amplification. The newly obtained sequences were deposited into the GenBank database (accession numbers: MW346650 for the SSU sequence of the new species, MW346649 for the LSU of the new species; MW346646, MW346647, MW346648 for the LSU sequences of *Malenchus gilanensis*).

### Phylogenetic analyses

The newly obtained SSU and LSU sequences were compared with those of other nematode species available in GenBank using the BLAST homology search program. The selected DNA sequences (for species and accession numbers, see SSU and LSU trees) for inferring the SSU and LSU phylogenies were aligned using ClustalX2 (http://www.clustal.org/) and the resultant alignments were manually edited using MEGA6 (Tamura et al., 2013). The model of base substitution was selected using MrModeltest 2 (Nylander, 2004). The Akaike-supported model, a general time reversible model, including among-site rate heterogeneity and estimates of invariant sites (GTR +G + I) was selected and used in both phylogenies. The Bayesian analyses were performed using MrBayes v3.1.2 (Ronquist and Huelsenbeck, 2003) and a random starting tree, running the chains for 5 × 10^6^ generations for both datasets. After discarding burn-in samples, the remaining samples were retained for further analyses. The Markov chain Monte Carlo (MCMC) method within a Bayesian framework was used to estimate the posterior probabilities of the phylogenetic trees (Larget and Simon, 1999) using the 50% majority rule. Convergence of model parameters and topology were assessed based on average standard deviation of split frequencies and potential scale reduction factor values. Adequacy of the posterior sample size was evaluated using autocorrelation statistics as implemented in Tracer v.1.6 (Rambaut and Drummond, 2009). The outgroup taxa of both trees were selected according to previous studies (Gharahkhani et al., 2020; Panahandeh et al., 2019c). The output ﬁles of the trees were visualized using Dendroscope v3.2.8 (Huson and Scornavacca, 2012) and drawn in CorelDRAW software version 12.

## Results

### *Filenchus pseudodiscus* n. sp.

**Figure 1:**
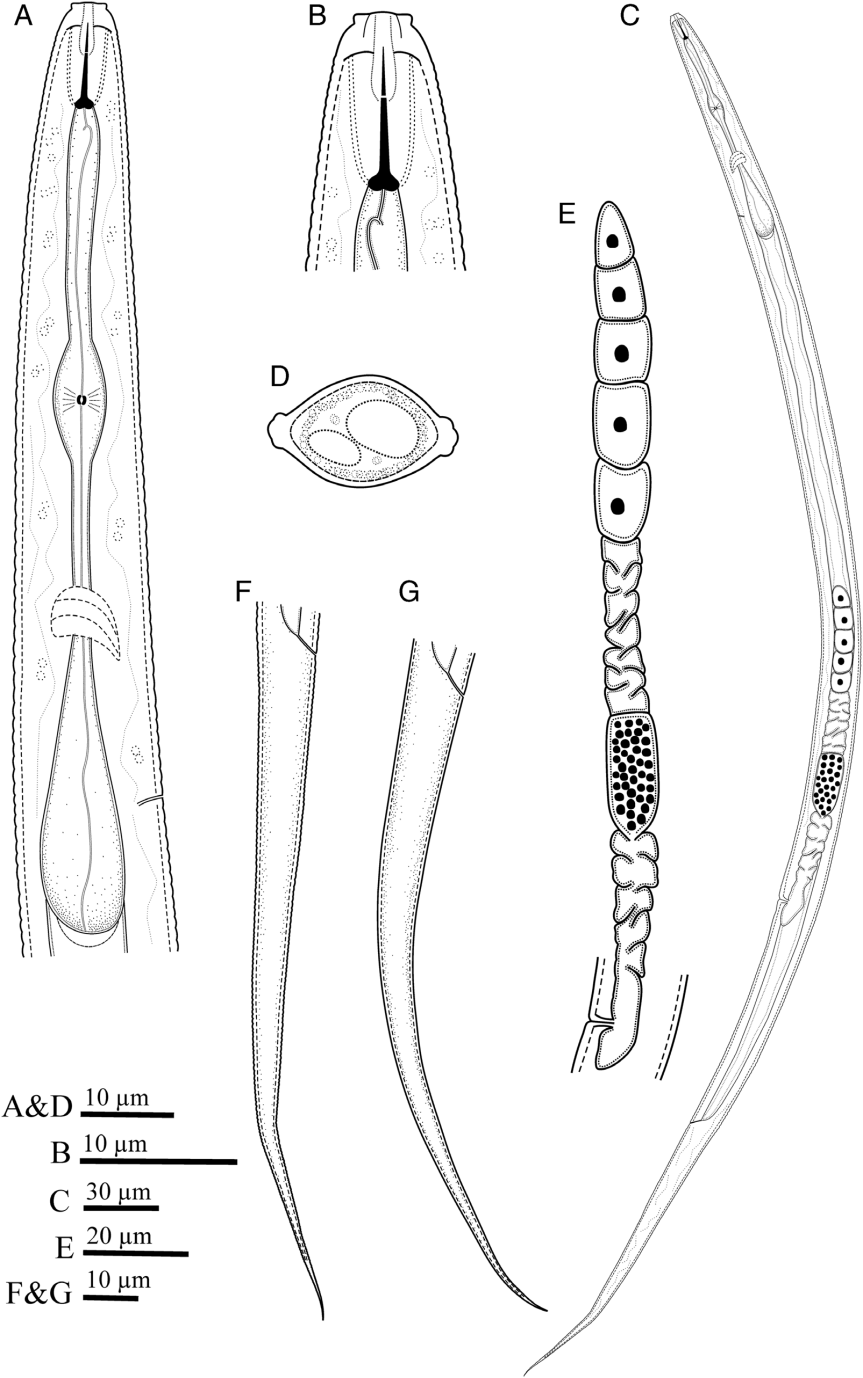
Line drawings of *Filenchus pseudodiscus* n. sp. (female). (A) Pharyngeal region. (B) Anterior end. (C) Entire body. (D) Cross section. (E) Reproductive system. (F, G) Tail.

**Figure 2:**
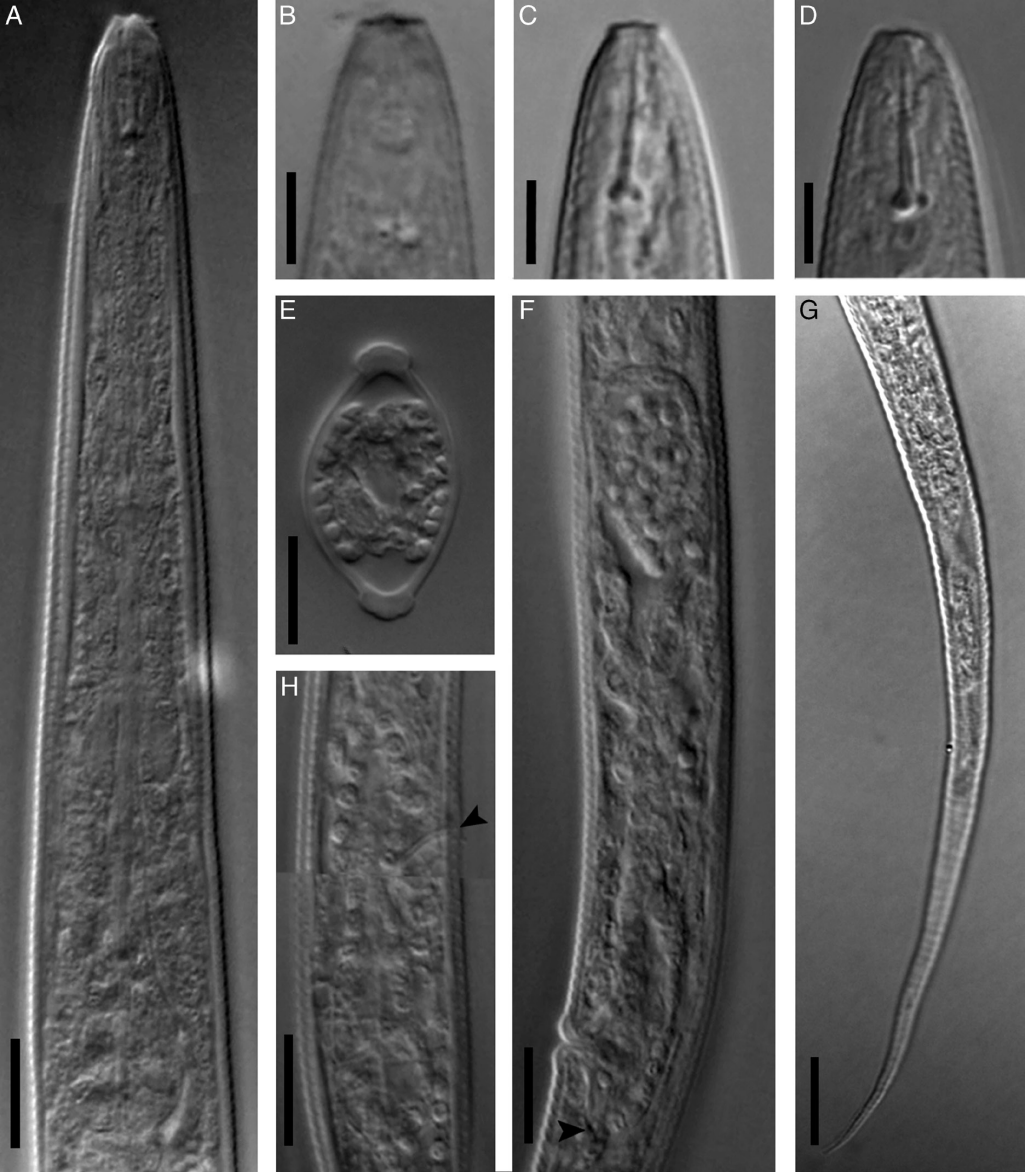
Light microphotographs of *Filenchus pseudodiscus* n. sp. (female). (A) Pharynx. (B) Anterior end showing cephalic region. (C, D) Anterior end showing cephalic region and stylet (fresh material in water). (E) Cross section at mid-body. (F) Part of female reproductive system (arrowhead showing end of PUS). (G) Tail. (H) Pharyngeal bulb region (arrowhead showing excretory pore). (A, E-H = 10 µm, B-D = 5 µm.)

**Figure 3:**
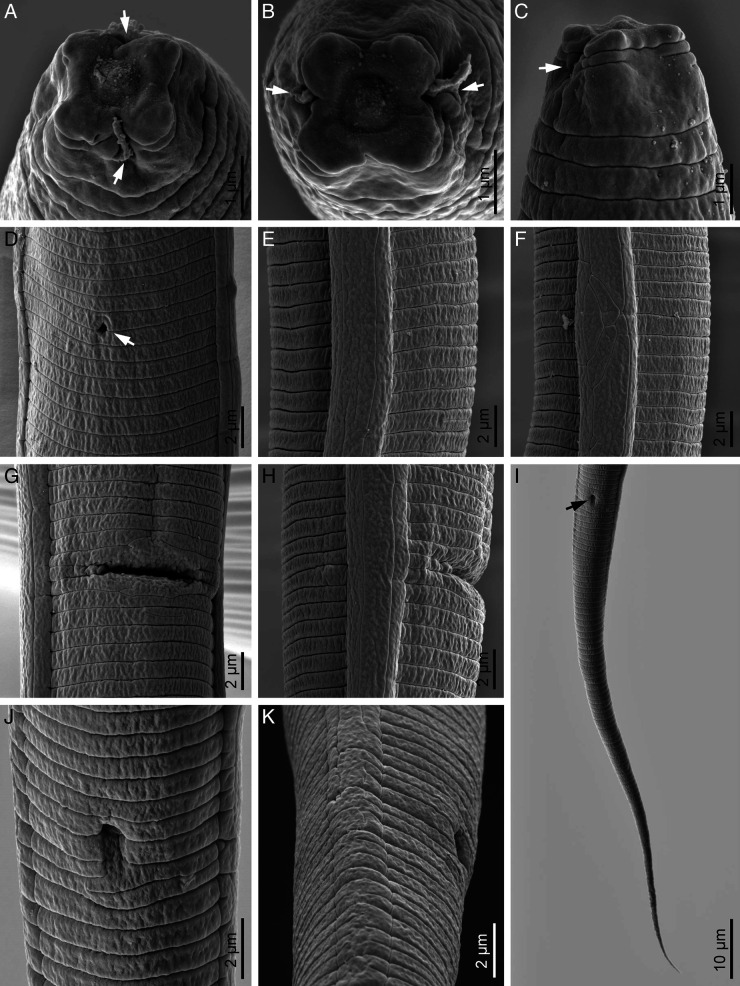
Scanning electron microphotographs of *Filenchus pseudodiscus* n. sp. (female). (A-C) Cephalic region in latero-frontal, frontal and subventral views, respectively (arrows pointing the amphidial openings). (D) Excretory pore in ventral view (arrow). (E, F) Lateral field at mid-body showing four incisures and unusual division, respectively. (G, H) Vulva in ventral and lateral views, respectively. (I) Posterior end (arrow pointing the anus). (J, K) Anus in ventral and lateral views, respectively.

**Figure S1:**
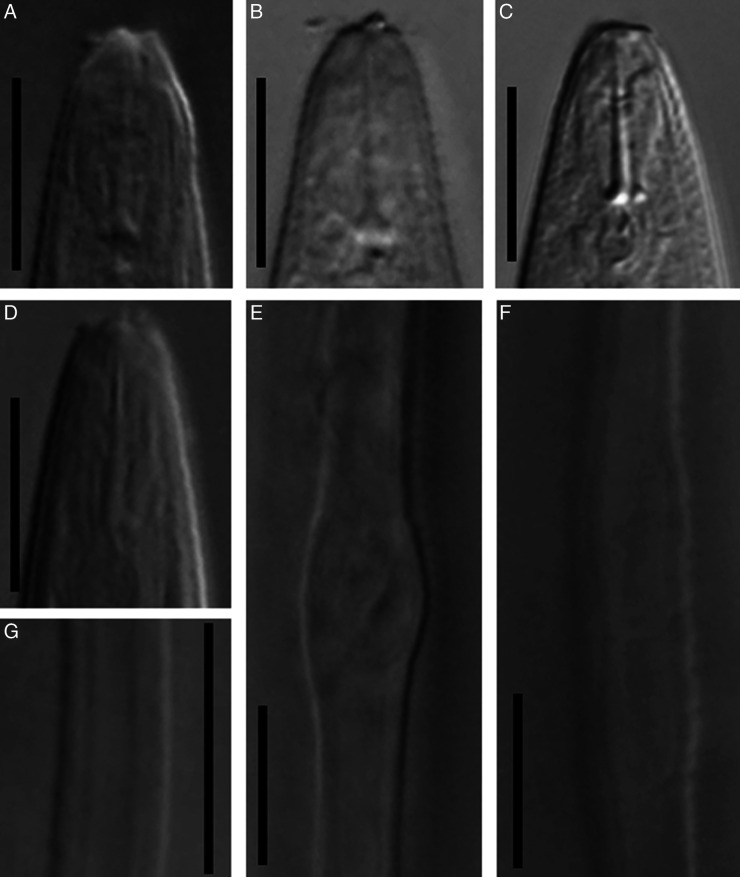
Light microphotographs of *Filenchus pseudodiscus* n. sp. (female). (A, B) Anterior end showing stylet. (C, D) Anterior end showing stylet and DGO. (E, F) Irregular lateral lines forming a swollen portion. (G) Lateral field at mid body showing four incisures. (All scale bars = 10 μm).

(Table 1; Figs. 1-3; Supplementary Fig. 1).

**Table 1.** Morphometrics of *Filenchus pseudodiscus* n. sp. and Golestan province population of *Malenchus gilanensis* (Jalalinasab et al., 2019).

	*Filenchus pseudodiscus* n. sp.		*Malenchus gilanensis*	
	Holotype	Paratypes	Males	Females
*n*	1 female	10 females	2	8
*L*	593	586 ± 22.3 (555-618)	268, 290	318 ± 16.8 (296-341)
*a*	39.5	37.0 ± 2.4 (30.8-39.5)	26.8, 36.3	28.7 ± 3.2 (25-34)
*b*	6.1	5.8 ± 0.3 (5.5-6.3)	4.1, 3.6	4.0 ± 0.3 (3.6-4.3)
*c*	4.7	4.7 ± 0.1 (4.5-4.8)	5.7, 5.9	6.9 ± 0.5 (6.0-7.7)
*c΄*	12.6	11.8 ± 0.7 (10.9-12.8)	4.7, 8.9	7.2 ± 1.1 (6-9)
*V*	63	63.1 ± 0.8 (62-64)	33.6, 29.3	66.8 ± 4.8 (64-76)
Cephalic region height	2	2.4 ± 0.2 (2-3)	2.5, 3.0	2.0 ± 0.5 (2-3)
Cephalic region width	5	5.1 ± 0.3 (5-6)	5, 5	5.0 ± 0.5 (4-5)
Stylet length	10	9.6 ± 0.4 (9-10)	7.2, 8.0	7.5 ± 0.5 (7-8)
Conus length	3	3.3 ± 0.3 (3-4)	3.5, 4.0	2.5 ± 0.6 (3-4)
m	34	34.3 ± 1.6 (32-36)	36.8, 42.5	37.8 ± 2.0 (35.5-41.9)
Dorsal gland opening	1	1.4 ± 0.3 (1-2)	–	–
Median bulb from ant. end	42	43.0 ± 2.3 (40-46)	–	–
MB	43	42.5 ± 2.7 (39-47)	–	–
Excretory pore to ant. end	80	80.3 ± 6.0 (72-94)	54, 60	60.8 ± 3.8 (56-65)
Pharynx length	98	101.2 ± 3.3 (97-107)	65, 80	81.5 ± 3.9 (75-86)
Cephalic region to vulva	375	370.3 ± 14.8 (350-389)	–	211 ± 11 (197-226)
Body width	15	15.9 ± 1.3 (15-19)	15, 18	11.0 ± 1.4 (9-13)
Tail length	126	125 ± 5 (115-133)	47, 49	45 ± 5 (42-55)
Tail/vulva-anus	1	1.4 ± 0.1 (1.2-1.5)	–	–
Anal body width	10	10.6 ± 0.5 (10-11)	–	7.4 ± 1.0 (6-9)
Post-vulval uterine sac	8	10.0 ± 1.5 (8-12)	–	6.3 ± 0.8 (5-7)

**Note:** All measurements are in μm and in the form: mean ± s.d. (range).

## Description

### Female

Body slightly ventrally curved after heat relaxation. Annuli fine but distinct. Lateral ﬁelds with four incisures in cross section (Fig. 2E), lateral view under LM (Supplementary Fig. 1G) and SEM photos (Fig. 3E, H). In some specimens, there is a slightly wider portion on lateral field at 62 to 80 μm distance from vulva toward anterior body with irregular arrangement of lateral lines on only one side of the body. This portion is characterized by irregular lines forming about eight differently sized blocks (Fig. 3F and Supplementary Fig. 1E, F). Cephalic region continuous with the body, 2 to 3 μm high and 5 to 6 μm broad at base, having a disc-like structure at apex with 3 to 4 diameter under LM. SEM images showing a high, smooth cephalic region, lacking a true disc at apex, having one narrow annulus behind cephalic plate, the smooth region behind this annulus about twice body annuli wide, the amphidial apertures as elongated slits, starting behind cephalic plate, extending into anterior portion of the smooth region, the cephalic plate is four-lobed, includes four vestigial cephalic sensilla in the shape of shallow pits at corners of each lobe, and a small rounded oral aperture encircled by six sensilla. Stylet moderately developed, conus *ca* 34% of its total length, with posteriorly directed knobs. Dorsal gland orifice (DGO) 1 to 2 μm from base of stylet. Pharynx tylenchoid, the procorpus joining to an oval metacorpus with a small valvular apparatus, isthmus slender, and basal bulb saccate. Excretory pore at the nearly midway between nerve ring and basal bulb. Hemizonid not apparent. Reproductive system monodelphic-prodelphic, composed of an outstretched ovary with the oocytes in a single row, tubular oviduct, elongated-oval offset spermatheca filled with spheroid sperm, the detail of the structure of crustaformeria not clearly seen, uterus thick-walled, vagina straight, vulva a transverse simple slit and PUS 0.5 to 0.6 times vulval body width long. Rectum and anus functional. Tail conical, uniformly tapering, with pointed end.

### Male

Unknown.

### Type habitat and locality

The new species was recovered from a soil sample collected from the rhizosphere of *Quercus* sp. in a natural forest close to the city of Minudasht, Golestan province, north Iran during 2018. GPS coordinates: N37°09.323′, E55°21.108′.

### Type material

Holotype female and ten paratype females were deposited at the Nematology Collection of Faculty of Agriculture, Tarbiat Modares University, Tehran, Iran.

### Etymology

The specific epithet refers to the disc-like differentiation in frontal end of cephalic region of the new species under LM.

### Diagnosis and relationships

The new species is mainly characterized by its disc-like differentiation at frontal end under LM, smooth cephalic region under SEM and a narrow annulus behind the cephalic plate (Fig. 3C). It is further characterized by having a squarish four lobed cephalic plate including vestigial cephalic papillae at corners, short longitudinal amphidial slits under SEM, lateral fields with four incisures, offset elongate-ellipsoid spermatheca filled with spheroid sperm, short PUS and elongate uniformly tapering tail with pointed tip. By lacking a true disc in frontal end (also see Discussion), and having a squarish cephalic plate with rounded corners, the new species was assigned to the genus *Filenchus* (Geraert, 2008); and by having a disc-like structure in frontal end under LM, proposing it as a tentative member of *Discotylenchus*, the new species was morphologically compared with relevant species (i.e. having four lines in lateral fields) of both aforementioned genera.

In comparison with the relevant species of *Filenchus* having four lines in the lateral fields and similar general morphology, the new species differs by having a disc-like differentiation in frontal end under LM (except *F. cylindricaudus* (Siddiqi, 1986; Wu, 1969) having a similar structure of cephalic region). The detailed comparisons are as follow:

From *F. andrassyi* (Szczygieł, 1969) by having a moderately developed stylet with distinct knobs (vs thin, with very small knobs) and not hooked tail end (vs hooked), a shorter body (586 (555-618) vs 620-930 μ m) and greater *c′* (11.8 (10.9-12.8) vs 5.9-9.9).

From *F. cylindricaudus* by having distinct body annuli (vs indistinct), moderately developed stylet (vs thin, after the drawings), a shorter body (586 (555-618) vs 860-950 µ m), shorter stylet (9.6 (9-10) vs 11.0-12.5 μm), smaller *a* (37.1(30.8-39.5) vs 41-46), smaller *c* (4.7 (4.5-4.8) vs 6.2-7.0) and anteriorly located vulva (*V* = 63.1 (61.9-64.1) vs 66-67).

From *F. orbus* (Andrássy, 1954) by having an oval median bulb (vs thin), shorter body (586 (555-618) vs 720-790 µ m) and stylet (9.6 (9-10) vs 12.0-12.5 µ m) and smaller *c* (4.7 (4.5-4.8) vs 6.6-7.4).

From *F. quartus* (Szczygieł, 1969) by having an elongated-oval spermatheca (vs rounded), shorter stylet (9.6 (9-10) vs 11.5-12.5 µ m) and shorter tail (125 (115-133) vs 142-191 µ m).

From *F. vulgaris* (Brzeski, 1963) by having a smooth cephalic region with a one narrow annulus behind the cephalic plate (vs 4-5 delicate annuli), moderately developed stylet (vs delicate) and conical uniformly tapering tail having a pointed tip (vs gradually tapering to a very thin filamentous posterior part).

By having a disc-like differentiation under LM, the new species was compared with three known relevant species of *Discotylenchus* (also see Discussion) having four lines in the lateral ﬁelds, namely *D. attenuatus* (Siddiqi, 1980), *D. biannulatus*, and *D. brevicaudatus* (Brzeski, 1985) as follow:

From *D. attenuatus* by having a longer body (586 (555-618) vs 330-400 µm), stylet (9.6 (9-10) vs 6.0-6.5 µm) and tail (125 (115-133) vs 68-96 µm).

From *D. biannulatus*, by having distinct body annuli (vs fine), shorter body length (586 (555-618 µm) vs 691 (675-708 µm)), smaller *c′* (11.8 (10.9-12.8) vs 15.1 (14.1-16.1)), posteriorly located vulva (*V* = 63.1 (61.9-64.1) vs 60.1 (59.5-60.6)) and shorter tail (125 (115-133) vs 158.2 (155-161)).

From *D. brevicaudatus*, by having a longer body (586 (555-618) vs 320-360 µ m) and stylet (9.6 (9-10) vs 7-8 µ m), smaller *c* (4.7 (4.5-4.8) vs 8.1-9.8), greater *c′* (11.8 (10.9-12.8) vs 4.5-6.0), anteriorly located vulva (*V* = 63.1 (61.9-64.1) vs 71-73), longer tail (125 (155-133) vs 35-77 µm), and tail with pointed end (vs thick with rounded end).

### Golestan province population of *Malenchus gilanensis*

(Table 1; Fig. 4).

**Figure 4:**
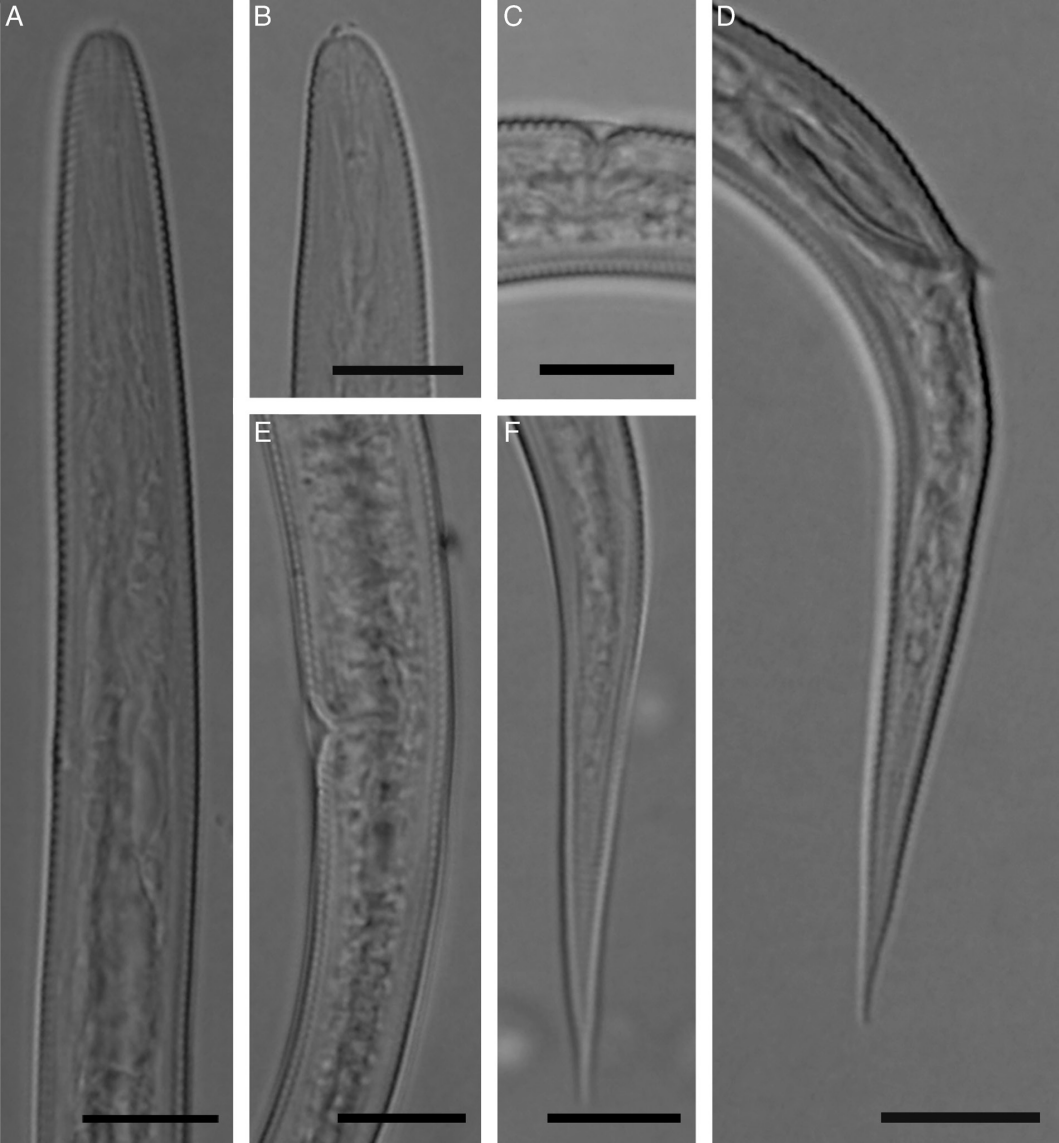
Light microphotographs of Golestan province population *Malenchus gilanensis* (Jalalinasab et al., 2019). (A) Pharynx. (B) Cephalic region and stylet. (C) Vulva region showing lateral flap. (D) Tail and spicule in lateral view. (E) Vulva region showing post-vulva uterine sac. (F) Female tail. (All scale bars = 10 μm).

The presently studied population of *Malenchus gilanensis* was recovered from Golestan province. It is in full morphological agreement with the type population; and no remarkable differences in morphology and the range of morphometric data were observed in comparison with the type population described from Gilan province (Jalalinasab et al., 2019).

### Molecular phylogenetic status

The amplification and sequencing of SSU and LSU rDNA (D2-D3 region) fragments of the new species yielded single fragments of 788 and 726 nt long (accession numbers MW346650 and MW346649, respectively). The BLAST search using the SSU sequence, revealed that its highest identity (99.37%) with currently available SSU sequences of Tylenchidae belonged to *F. vulgaris* (KJ869307) and *Filenchus* sp. (JQ814877). However, the BLAST search using the LSU sequence revealed that its identity with all currently available LSU sequences of Tylenchidae is less than 93% (the highest identity was 92.21%, belonging to *F. vulgaris*, MN542187). The BLAST search using the newly generated LSU sequences of *Malenchus gilanensis* (accession numbers MW346646-MW346648) revealed their identity with currently available sequences was less than 86%. To determine the phylogenetic affinities of the new species with other genera/species of the family Tylenchidae, SSU, and LSU datasets were compiled.

**Figure 5:**
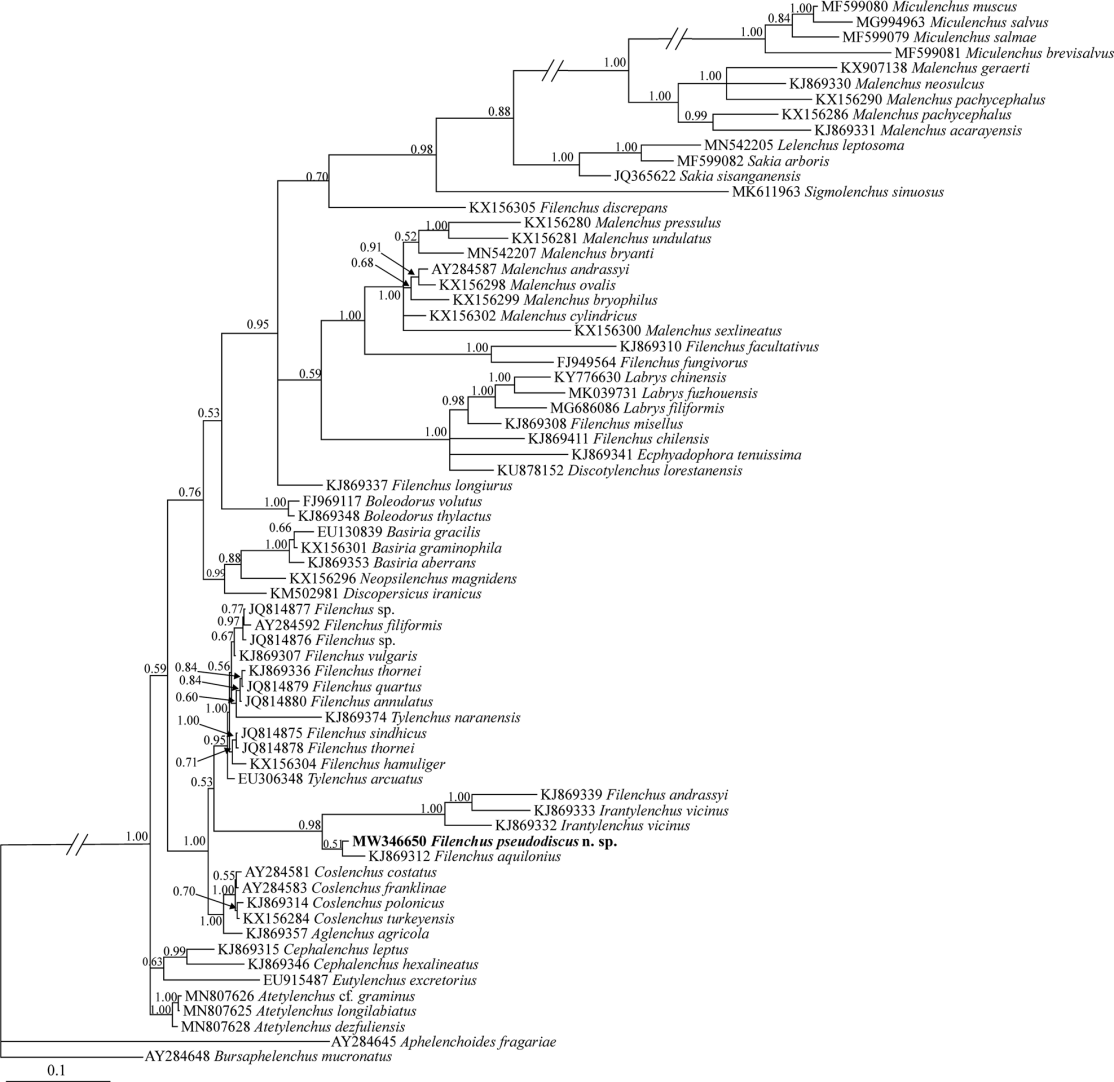
Bayesian 50% majority rule consensus tree inferred from SSU rDNA of *Filenchus pseudodiscus* n. sp. under the GTR+G+I model. Bayesian posterior probabilities (BPP) more than 50% are given for appropriate clades. The new sequence is in bold font.

The SSU dataset included 69 sequences (including newly generated sequence of the new species and two aphelenchoidid sequences as outgroups). Figure 5 represents the Bayesian phylogenetic tree inferred using this dataset. In this tree, several species of *Filenchus* have occupied different placements. The new species was clustered into a clade including representatives of Tylenchinae, with *F. aquilonius* (Wu, 1969) (KJ869312) being its closest relative.

**Figure 6:**
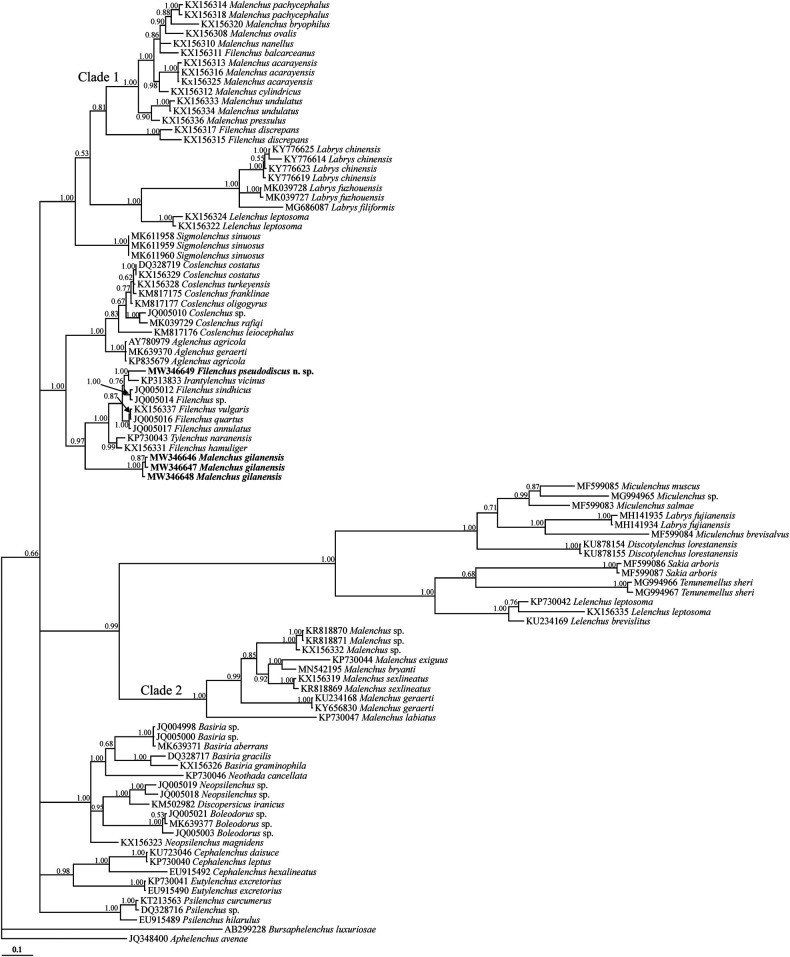
Bayesian 50% majority rule consensus tree inferred from LSU rDNA D2-D3 of *Filenchus pseudodiscus* n. sp. and *Malenchus gilanensis* under the GTR + G + I model. Bayesian posterior probabilities (BPP) more than 50% are given for appropriate clades. New sequences are in bold font.

The LSU dataset was composed of 98 sequences (including newly generated sequence of the new species, three newly generated sequences for *Malenchus gilanensis* and two aphelenchid and aphelenchoidid sequences as outgroups). The inferred tree using this dataset is presented in Figure 6. In present tree, several species of *Filenchus* have occupied different placements. The new species was clustered into a clade including representatives of Tylenchinae. The three sequences of *Malenchus gilanensis* are in sister relation to the aforementioned clade including the new species.

## Discussion

In the present study, one new species of the family Tylenchidae was described and illustrated based upon morphological, morphometric, and molecular characters. It was assigned to the genus *Filenchus* based upon detailed SEM observations on the cephalic region. A real disc in frontal end, as appeared under LM, did however not exist (see below). In accordance with some recent phylogenies using ribosomal markers (e.g. Panahandeh et al., 2018; Qing and Bert, 2018), the two currently resolved phylogenies using both SSU and LSU ribosomal markers, show the genus *Filenchus* is polyphyletic. These results are updates to the study of Atighi et al. (2013), showing *Filenchus* is polyphyletic using SSU, but monophyletic using LSU data.

Currently there are six species under the genus *Discotylenchus* (Geraert, 2008) all of which being established based upon morphological criteria. The SEM data are however not available for their type populations. The only currently available SEM data for a representative of the genus, belong to those given for an Iranian population of *D. discretus* presented by Yaghoubi et al. (2016), clarifying the status of what is named a ‘disc’ in this species under LM. Thus, the detailed structure of the so called ‘disc’ is unknown for the rest five species under the genus listed by Geraert (2008). Such an appearance of a ‘disc’ or a ‘disc-like structure’ under LM, recently gave rise to adding another species, *D. biannulatus*, to the genus (Konani et al., 2018). Based on both morphological observations and molecular data on the generic status of the new species, and with regarding the similar cephalic region structure of *F. cylindricaudus* already clarified using SEM images by Karegar and Geraert (1998), the generic status of *Discotylenchus biannulatus* needs additional data (it might better to be moved to *Filenchus*). It is however proposed that in lacking SEM data, the species with typological similarities to both genera *Filenchus* and *Discotylenchus* and having a disc-like structure, need(s) to be compared with species under both genera. SEM studies (in integration with molecular data) of the tylenchid populations having disc-like differentiation in frontal end plus additional characteristics gave rise to establishing Tylenchidae genera in recent years (Yaghoubi et al., 2016; Qing and Bert, 2018; Gharahkhani et al., 2020). Complementary SEM studies were also proposed for clarification the status of some species apparently erroneously assigned to other genera (see Panahandeh et al., 2019a for the status of *Discotylenchus lorestanensis* Mehrabian et al., 2017).

There are now two well-accepted subgenera under *Malenchus*. The subgenus *Malenchus* (*Malenchus*) and the subgenus *Malenchus* (*Telomalenchus*) (Geraert, 2008; Qing and Bert, 2017). The former is characterized by having a zigzag appearance of amphidial slit and a lateral line including up to 12 finer lines (only visible under SEM). The latter subgenus has longitudinal amphidial slits and four or six lateral fields. *Malenchus gilanensis* has longitudinal amphidial slits, making it similar to the subgenus *Malenchus* (*Telomalenchus*), but a single lateral line, similar to that found in subgenus *Malenchus* (*Malenchus*) (Jalalinasab et al., 2019). Based upon previous phylogenies of *Malenchus* spp. (e.g. Pedram et al., 2018; Qing et al., 2017), the genus *Malenchus* (using currently available data for the subgenus *Malenchus* (*Malenchus*), as there are currently no molecular data for the representatives of *Malenchus* (*Telomalenchus*)), was shown to be monophyletic in SSU phylogeny. In recent phylogenies (e.g. Panahandeh et al., 2019c; Bai et al., 2020; present study) including the new genera and species, showed the genus is paraphyletic in SSU phylogeny. In LSU phylogeny, however, the species are divided into two major clades: the clade1 + *Filenchus balcarceanus* (Torres and Geraert, 1996) (a species with similar appearance to *Malenchus* spp., based upon its cuticle); and the clade 2 (e.g. Pedram et al., 2018; Qing et al., 2017). The present LSU phylogeny however is an update to the previously resolved phylogenies for *Malenchus*, showing the currently available LSU sequences of the genus form three major groups (two aforementioned major clades plus the clade of *Malenchus gilanensis* + some Tylenchidae species), corroborating a real polyphyletic status for it (after currently available data).
